# Laser Processing of Diffusion Boronized Layer Produced on Monel^®^ Alloy 400—Microstructure, Microhardness, Corrosion and Wear Resistance Tests

**DOI:** 10.3390/ma14247529

**Published:** 2021-12-08

**Authors:** Aneta Bartkowska, Dariusz Bartkowski, Damian Przestacki, Mateusz Kukliński, Andrzej Miklaszewski, Piotr Kieruj

**Affiliations:** 1 Institute of Materials Science and Engineering, Faculty of Materials Engineering and Technical Physics, Poznan University of Technology, ul. Jana Pawła II 24, 61-138 Poznan, Poland; andrzej.miklaszewski@put.poznan.pl; 2 Institute of Materials Technology, Faculty of Mechanical Engineering, Poznan University of Technology, ul. Piotrowo 3, 61-138 Poznan, Poland; dariusz.bartkowski@put.poznan.pl; 3 Institute of Mechanical Technology, Faculty of Mechanical Engineering, Poznan University of Technology, ul. Piotrowo 3, 61-138 Poznan, Poland; damian.przestacki@put.poznan.pl (D.P.); mateusz.kuklinski@doctorate.put.poznan.pl (M.K.); piotr.kieruj@put.poznan.pl (P.K.)

**Keywords:** diffusion boronized layer, laser processing, Monel, microstructure, microhardness, chemical composition, phase composition, corrosion resistance, wear resistance

## Abstract

The paper presents the results of studies of microstructure, mechanical and physicochemical properties of surface layers produced by laser modification of the diffusion boron layer on Monel^®^ Alloy 400. The diffusion boron layers were produced at 950 °C for 6 h. The gas-contact method was used in an open retort furnace. The process was carried out in a powder mixture containing B_4_C carbide as a boron source. The next stage was the modification of the boron layer with a diode laser beam of a nominal power of 3 kW. A constant power of 1400 W of the laser beam was used. The scanning speed was variable (successively 5 m/min, 25 m/min, 50 m/min). In order to determine the best parameters, single tracks were created, after which multiple tracks were prepared using previously selected parameters. It was found that both the diffusion borided layer and the laser modified layer had better properties than the substrate material. Both these processes contributed to an increase in corrosion resistance, hardness and wear resistance. It was also found that laser modification caused a slight deterioration of the properties in comparison with the diffusion borided layer. However, the laser modification process resulted in the production of a much thicker layer.

## 1. Introduction

Nickel alloys are widely used in the automotive and chemical or marine industries. A representative of such alloys is Monel^®^ Alloy 400, but its low hardness and wear resistance restrict the fields in which it can be applied [1,2]. Although it has good corrosion resistance, mechanical properties are undoubtedly important. Monel^®^ Alloy 400 is a Ni-Cu alloy known for its high corrosion resistance, in particular in seawater, alkaline solutions, salts and selected acids such as organic, sulfuric, hydrofluoridic and phosphoric. These alloys are widely used in valves, pumps, heat exchangers, bearings and shafts, also working in the equipment of marine and oil industry. Monel^®^ Alloy 400 does not show good results in terms of wear resistance due to its low hardness, which disqualifies this material from applying where it would be exposed to wear, erosion, cavitation or adhesive wear. There are many methods of alloy surface modification. Interesting processes of improving properties of the surface layer in terms of obtained properties are diffusion and laser processes. On the other hand, the element that contributes to unique properties is boron [3,4,5]. Boron introduced by diffusion method contributes to the formation of a needle-like structure of boron, which is characterized by high hardness and good wear resistance as well as corrosion resistance. However, a drawback that may occur in this type of layer is its brittleness in the subsurface zone, which may be manifested by chipping of the formed layer and possible cracks. Therefore, in this study, an additional step was taken in the modification of such a layer and laser processing was used. The use of this process aims to obtain a layer with an even distribution of boron in the layer. In addition, boron mixing with the base material contributes to obtaining a layer with new unique properties, probably without the aforementioned drawbacks in the form of brittleness. To the authors’ best knowledge, reports focused on surface modification Monel 400 by boron for improving the mechanical properties are very rare in the literature.

The diffusion boriding process is quite common, however, a majority of reports in literature concern modification of iron alloy substrates. Many authors have noticed a beneficial effect of laser beams on the boron layer, which gained new unique properties. Often, better properties were achieved than for layers produced in diffusion processes [6,7,8,9]. Appropriate selection of production parameters contributed to obtaining very good mechanical properties, such as a favorable hardness profile from the surface into the material [6,7,8,9]. The operational parameters were also improved, especially resistance to friction wear, which ensures a long service life of the product [8,9]. Understanding the impact of laser beams on borides formed by diffusion processes in ferrous alloys initiated research on the modification of diffusion-formed borides on other groups of materials. Similar tests have been carried out on nickel alloys and have been described in the literature. Most of the publications on the production of a boron layer on nickel alloys concern materials such as Inconel [9,10,11,12,13,14], Nimonic [9,15] or pure nickel [15,16,17,18,19,20]. There are few reports in the literature on the modification of Ni-Cu alloys. In the publications that are available, the authors managed to find a single publication discussing diffusion boriding of Ni-Cu alloys [21]. Publications typically focus on the use of boron as a component of the additive material (powder) used in the laser cladding process (laser alloying [22,23,24,25], laser cladding [26,27]). In [27], a NiCrSiB coating was produced on Monel 400 using a laser cladding method, which was to improve resistance to cavitation erosion and corrosion. The authors demonstrated that by changing laser beam parameters, it is possible to obtain a hard, crack-free layer. The microstructure of the modified layer consists of cell dendrites and flake-like dendrites as well as eutectics. The modified layer is mainly a solid solution of Ni, chromium carbides (Cr_7_C_3_, Cr_23_C_6_) and Ni_3_B nickel borides. The authors showed that the obtained microstructure is characterized by a microhardness approximately 7 times greater than that of Monel 400 substrate. They also found that resistance to cavitation erosion and corrosion was significantly improved. In [22,23,24,25], the influence of boron introduced into the Ni-Cu alloy by means of a laser beam was studied; cracks at the border of laser tracks were found to be a certain drawback when creating a layer on Monel in laser boriding.

Due to a limited number of publications, and thus the novelty of the presented research on laser beam impact on boron-containing layer diffused into Monel^®^ Alloy 400 material, a study of this issue was undertaken. In literature, changes resulting from laser beam interaction on boron layer produced on Ni-Cu alloy are not currently discussed. Hence, it is worth getting acquainted with this issue and determining the changes caused by laser beam interaction with nickel borides produced on Monel^®^ Alloy 400.

## 2. Materials and Methods

Laser-modified, boronized surface layers were produced on Monel^®^ Alloy 400 material. The chemical composition of substrate material is given in Table 1. The specimens were in the form of plates with dimensions of 16 mm × 11 mm × 11 mm.

The diffusion boronized layers were produced in powder mixture consisting of boron carbides B_4_C, Al_2_O_3_, and AlF_3_. The boron source was B_4_C carbides (Sigma-Aldrich, Saint Louis, MI, USA). Kaolin played the role of filler, and aluminum fluoride was an activator. The diffusion boronizing process was carried out in a furnace with a so-called open retort. Specimens were placed in the pipe retort together with powder mixture, which was first preheated to set process temperature of 950 °C. Afterward, the retort was annealed for 6 h. When the diffusion boronizing process ended, the retort was cooled to room temperature. After diffusion, the specimens were cleaned from powder residues and degreased using acetone. The advantage of using the diffusion boriding process in comparison to the application of precoat in the form of paste [23,24,25] is the maintenance of a constant thickness on the entire surface of the specimen. In addition, the thickness of the diffusion layer can be controlled by extending or reducing the diffusion time.

**Figure 1:**
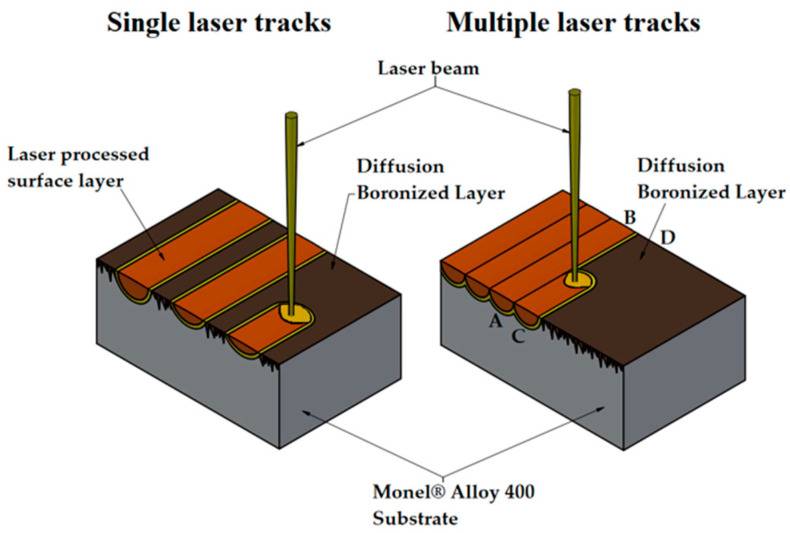
Scheme of laser processing of diffusion boronized layers produced on Monel^®^ Alloy 400 material both for single laser tracks and for multiple tracks.

In the next step, thus prepared specimens were subjected to laser processing. As a result of laser beam action, the diffusion boronized layer was mixed with the nickel-based alloy substrate, and as a consequence, the new surface layer was formed. Laser processing was performed on the 3 kW TruDiode 3006 diode laser (TRUMPF, Ditzingen, Germany). In these processes, constant laser beam power of 1400 W was applied. The laser beam scanning speeds varied and were as follows 5 m/min, 25 m/min, 50 m/min, respectively. The diameter of the laser beam was 1 mm and the transverse electromagnetic mode in this laser device was TEM_00_. The wavelength of the laser beam was 1040 nm. Applied laser beam power and the diameter of the laser beam gave that laser beam power density q = 178 kW/cm^2^. Laser tracks were produced separately and in each remelting laser beam was started from above and turned off behind the specimen. Based on the study of single tracks, the best parameter for multiple tracks was selected. Laser tracks were produced parallel to the longer side of the specimen, and after each process, the laser beam was shifted by f = 0.5 mm. The distance between the laser track axes was designed to obtain a level of overlapping of 50%. The schematic of the process for the single track as well as for multiple tracks is presented in Figure 1. The process of producing multiple tracks consisted of moving a laser beam from point A to B and then turning off the laser and returning from point B to point A. In the next step, the laser head was transferred by a distance of 0.5 mm from point A to point C, and then (after a turn of the laser) from point C to D. This activity was repeated until the entire surface of the specimen was laser modified.

Microstructure observations were carried out using an MIRA3 scanning electron microscope (TESCAN, Brno, Czech Republic) on cross-sections of specimen perpendicular to the produced surface layers. Prior to observation, all specimens were ground with papers with grit from 100 to 1500, polished using diamond paste and aluminum oxide, and finally etched in Marble’s solution for 10 s. The scanning electron microscope was equipped with an EDS-UltimMax energy dispersive spectrometer (Oxford Instruments, High Wycombe, UK) and dedicated Aztec Energy Live Standard software. During the EDS study 10 kV of energy was used, which corresponds to a penetration depth of approx. 0.3 μm. The phase composition of the specimens was analyzed using an EMPYREAN PANalytical X-ray diffractometer (Malvern Panalytical Ltd., Malvern, UK) operating in the range of 2θ = 20–90° by using Cu Kα radiation.

Microhardness tests were carried out on cross-sections of coatings both along and on the border of the laser tracks. This made it possible to find whether the obtained microhardness values are comparable on the entire newly formed layer produced and how the laser tracks affect each other. An FM-810 microhardness tester (Future-Tech, Kawasaki, Japan) equipped with an FT-Zero automatic indentation measuring software was used. Microhardness tests were made under an indentation load of 50 g, while the loading time was 15 s. 

Corrosion resistance tests were carried out using an ATLAS 1131 EU&IA device (At-las-Sollich, Rębiechowo, Poland) in 3.5% NaCl aqueous solution. The potentiodynamic method of anodic polarization curves was applied. The potentiodynamic measurements were performed at 22 °C, at a scanning speed of 1.0 mV/s. The reference electrode was a saturated calomel electrode, and the auxiliary electrode was a platinum electrode. Corrosion potential and corrosion current of analyzed specimens were determined in this study.

Wear resistance was tested on the plate-shaped specimens using an Amsler-type device (MBT, Poznan, Poland). The ring-shaped counter-specimens were made of CT90 tool steel after hardening from 780 °C in water and tempering at 180 °C for 1 h. The counter-specimens were 12 mm wide, inside diameter of 12 mm and outer diameter of 20 mm. Their hardness was 58 HRC. Wear resistance tests were performed in dry friction conditions using the following parameters: rotation speed of counter-specimen 250 rev/min, load 98 N, and friction time 60 min. The mass loss of specimens was measured using the AS220.R2 analytical balance (RADWAG, Radom, Poland) after every 10 min of wear test. The 3D surface topography and roughness profiles reconstruction after wear tests were determined on SEM images with Mountains^®^SEM software manufactured by Digital Surf company (Digital Surf Headquarters, Besançon, France).

## 3. Results and Discussion

### 3.1. Microstructure, Chemical and Phase Analysis

**Figure 2:**
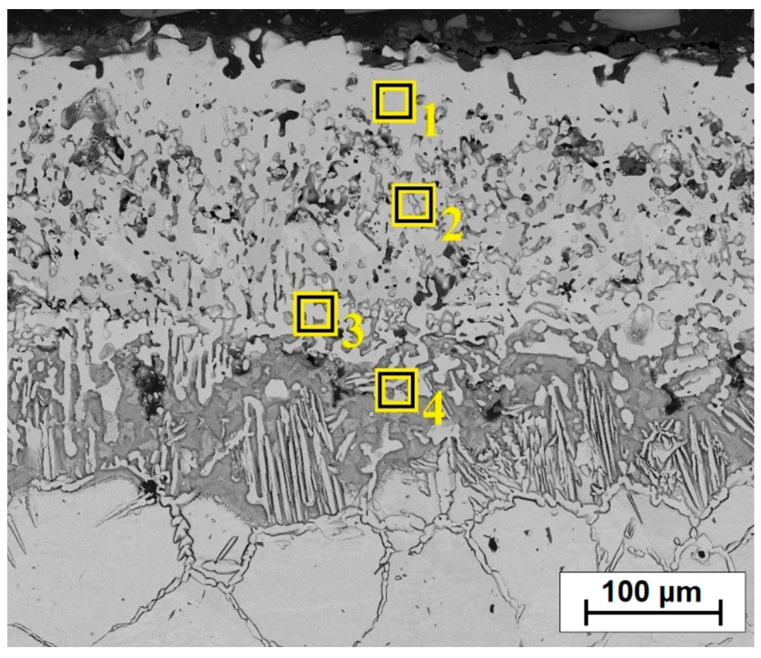
Microstructure of diffusion boronized layer with marked zones of EDS tests.

Figure 2 presents the microstructure of the boronized layers on Monel^®^ Alloy 400. A total thickness of the borided layer reached an average value of approximately 368 μm, while a visible continuous sub-surface zone had a thickness of 235 μm. Below the continuous zone, a boron layer formed on Monel^®^ Alloy 400 has a needle-like structure similar in nature to boron layers formed on iron alloys. It can also be seen that boron diffusion proceeded along grain boundaries of the nickel alloy, which can be seen quite well in the contrast of backscattered electrons (BSE). In papers where the authors subjected nickel alloys to diffusion boriding, different thicknesses of the surface layers were obtained depending on the amount of nickel and other elements in the alloy as well as the parameters of the boriding process (time and temperature). In paper [22], the authors found that with the increase of boriding time from 2 h to 6 h, the thickness of the boron layer on the Cu-Ni alloy increased from 50 µm to 90 µm. A similar relationship was observed for Inconel alloys. For this alloy type, the obtained thicknesses varied in relation to the parameters used and ranged from 54–74 µm [11] to 23–39 µm [10] respectively, for processing times from 2–6 h. Higher thickness values of borided layers were usually obtained by carrying out the process of pure nickel boriding. Özbek et al. [16] obtained a layer of 123 µm after 2 h of treatment at 950 °C, while Gunes and Kayali [14] achieved a thickness of 220 µm after 4 h at 900 °C.

The difference in the thickness of borided layers obtained by different researchers may result from two important causes, one of which is the presence of alloying elements in the substrate material. These elements can affect the possibilities and speed of diffusion of boron atoms. One such element that significantly influences boron diffusion is chromium. When chromium content is high, boron diffusion is inhibited, resulting in a thinner layer thickness at identical process durations and temperatures. The other important factor, which may also influence the obtained thickness of the borided layer is the chemical composition of powder mixtures used in the production of borided layers, containing SiC in their composition. Some researchers found that the formed layer of silicides is a barrier against boron atom diffusion [3,11,12]. However, in the studies of Ueda et al. [18] and Mu et al. [19], it was shown that there was a difference of only 20 μm in the thickness of the borided and borosilicate layers for the processes carried out at 900 °C for 2 h. Therefore, it is not possible to clearly indicate a significant influence of silicide layer formed, contrary to the interaction of other alloying elements from the substrate, e.g., chromium.

In [22], the authors obtained much thinner boron layers after 6 h on Monel 400 (of 90 µm) than in this study. However, it should be borne in mind that for the studies, they used commercial Ekabor 1-V2 powder (BorTec GmbH & Co. KG, Huerth, Germany) containing B_4_C as a boron source, KBF_4_ activator and SiC filler. In this study, the temperature reached 950 °C, and the activator was AlF_3_ and the filler was Al_2_O_3_. However, it should be mentioned that the nature of the obtained boron layer was very similar. Therefore, it can be concluded that the parameters of the diffusion boriding process (time and temperature of the process as well as boriding mixture composition) have a significant impact on the obtained layer thickness. The authors of the paper [22] used a substrate made of Monel 400 alloy, but with a slightly different chemical composition than in the studies presented in this article. As in the paper [22], the obtained borided layer (Figure 2) was characterized by the presence of small amounts of porosity in the sub-surface zone. Therefore, the next step was to modify the obtained boron layer on Monel^®^ Alloy 400 by laser processing. Three different laser beam scanning speeds were used with a constant laser beam power density. The first stage of the study was to create single laser tracks. Based on the observation of microstructure and microhardness measurements, the most favorable parameters for the second stage were determined. In the second stage, multiple tracks were made to analyze different properties such as friction wear resistance and corrosion resistance.

**Figure 3:**
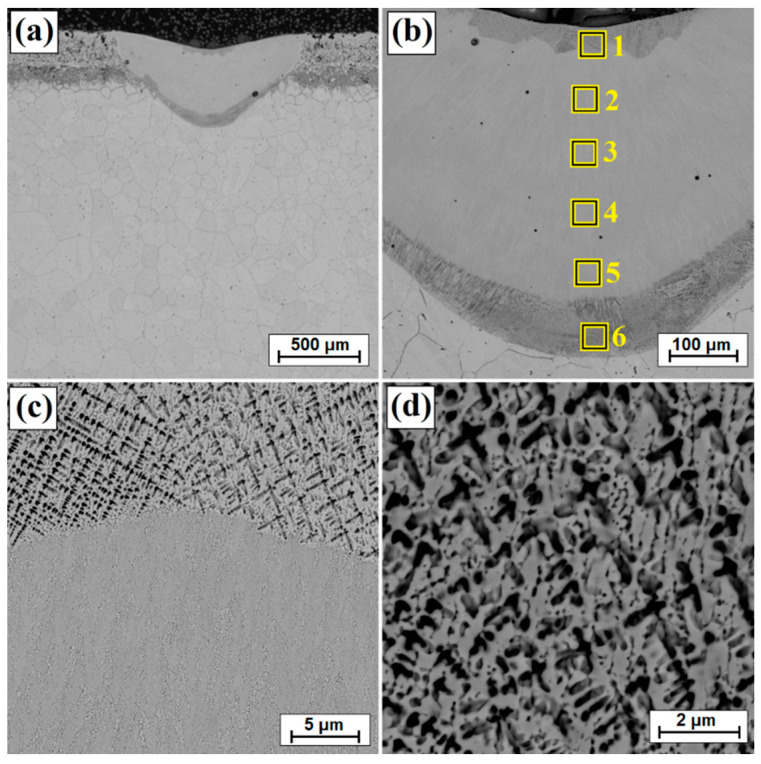
Microstructure of boronized layers after laser processing (v = 5 m/min)—single track: (**a**) morphology of laser track, (**b**) magnification of laser track, (**c**) subsurface zone of laser track, (**d**) magnification of subsurface zone of laser track.

**Figure 4:**
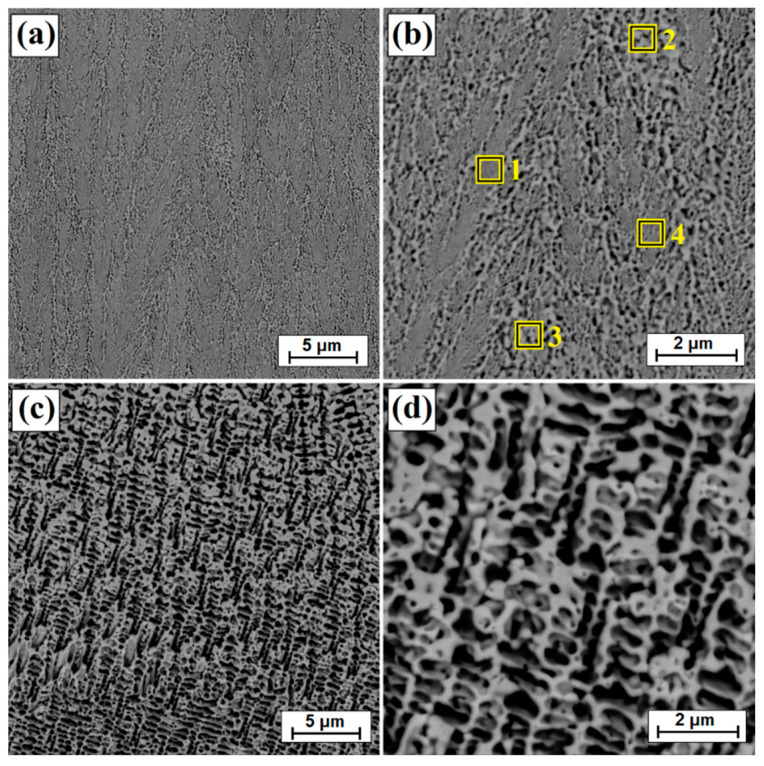
Microstructure of boronized layers after laser processing (v = 5 m/min)—single track: (**a**) middle zone of laser track, (**b**) magnification of middle zone of laser track, (**c**) sub-substrate zone of laser track, (**d**) magnification of sub-substrate zone of laser track.

**Figure 5:**
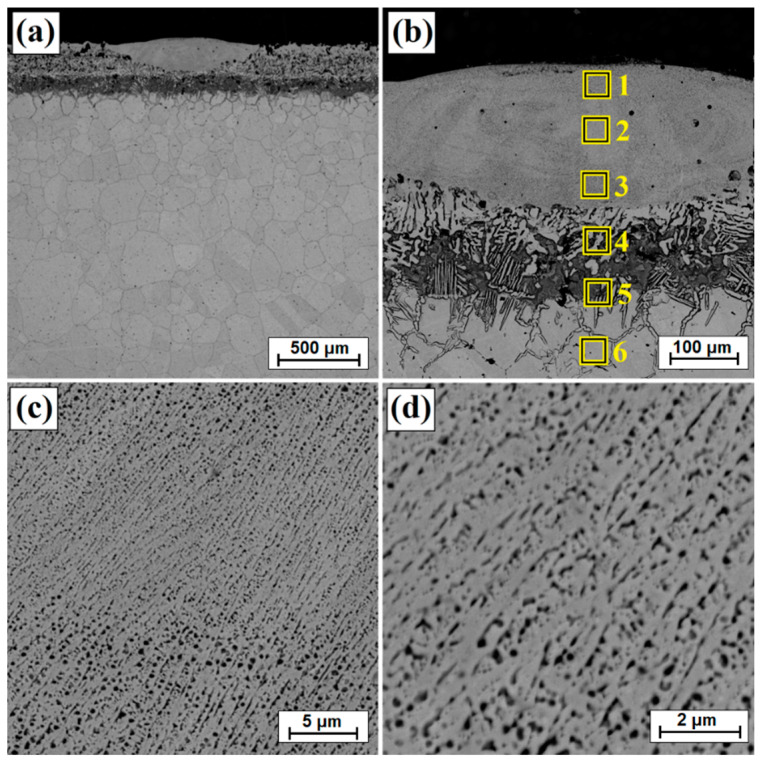
Microstructure of boronized layers after laser processing (v = 25 m/min)—single track: (**a**) morphology of laser track, (**b**) magnification of laser track, (**c**) subsurface zone of laser track, (**d**) magnification of subsurface zone of laser track.

**Figure 6:**
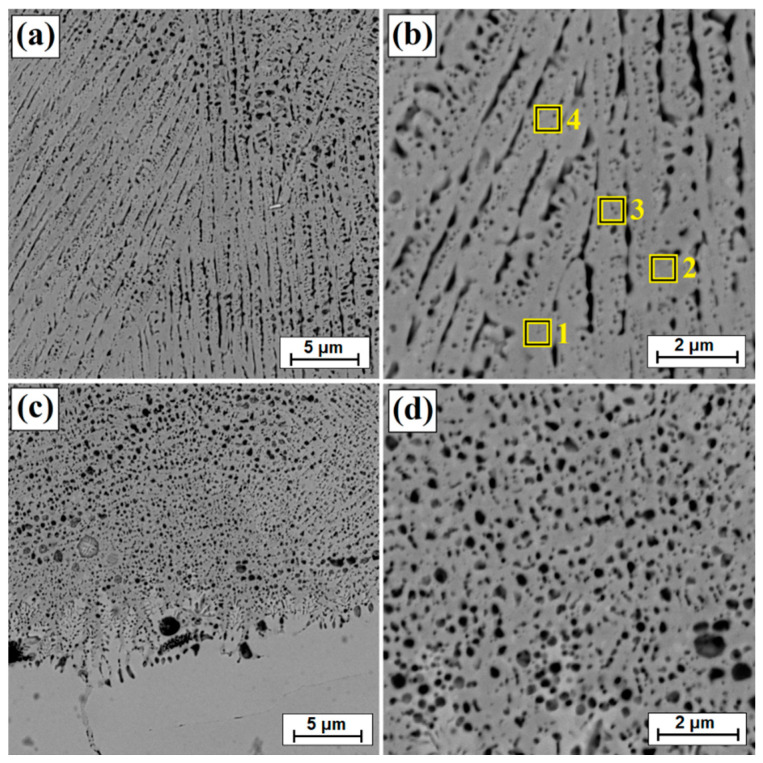
Microstructure of boronized layers after laser processing (v = 25 m/min)—single track: (**a**) middle zone of laser track, (**b**) magnification of middle zone of laser track, (**c**) sub-substrate zone of laser track, (**d**) magnification of sub-substrate zone of laser track.

**Figure 7:**
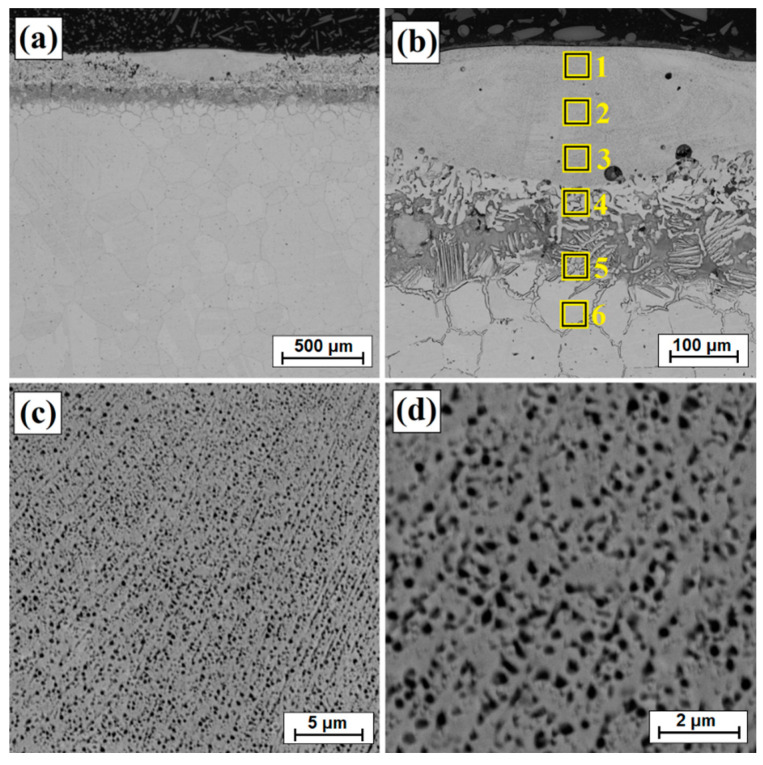
Microstructure of boronized layers after laser processing (v = 50 m/min)—single track: (**a**) morphology of laser track, (**b**) magnification of laser track, (**c**) subsurface zone of laser track, (**d**) magnification of subsurface zone of laser track.

**Figure 8:**
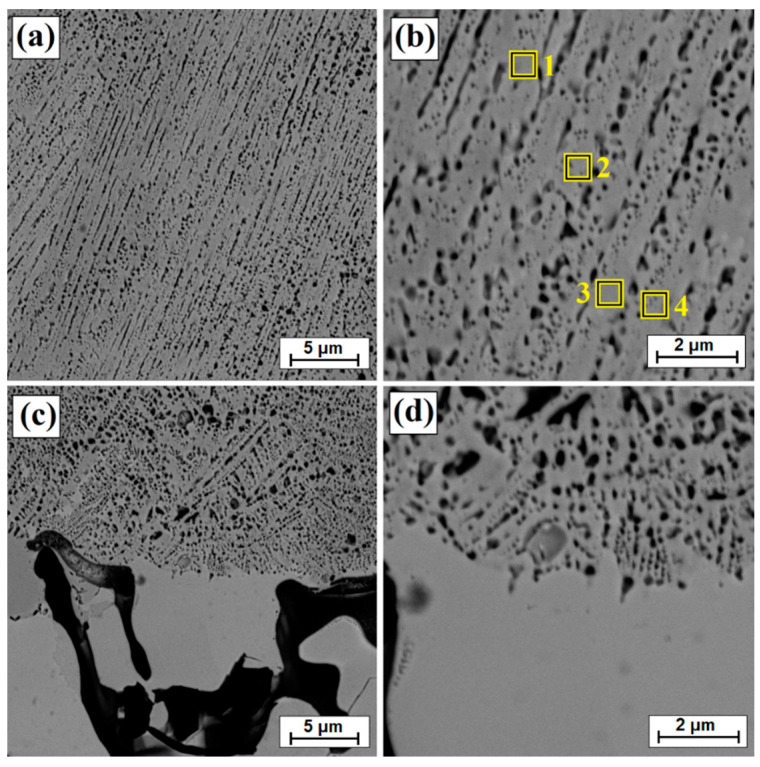
Microstructure of boronized layers after laser processing (v = 50 m/min)—single track: (**a**) middle zone of laser track, (**b**) magnification of middle zone of laser track, (**c**) sub-substrate zone of laser track, (**d**) magnification of sub-substrate zone of laser track.

Figure 3, Figure 4, Figure 5, Figure 6, Figure 7 and Figure 8 show the microstructure of diffusion borided layers after laser processing. As a result of laser beam impact at the lowest scanning speed (5 m/min), a laser track was obtained twice as thick as the diffusion borided layer. 

Figure 3b shows an enlarged area of the laser track from Figure 3a. A laser track was obtained consisting of a remelted zone in which it was possible to distinguish areas that had been otherwise affected by the chemical reagent used in etching. The subsurface, middle and sub-substrate areas of the laser track were analyzed. The magnification of these areas are shown in Figure 3c and Figure 4a,c, respectively; the obtained microstructure was fine-grained. In a significant part of the resulting laser track, the microstructure was even and homogeneous, consisting of a boron-nickel eutectic. At each site observed, the microstructure was dendritic. However, it was observed that in the upper part of the track and in the lower part at the border with the substrate, the dendrites were larger than in other areas. Figure 5 shows the microstructure of laser beam modified boron layer using a scanning speed of 25 m/min. A five times faster-scanning speed resulted in a laser track with a thickness comparable to the thickness of a diffusion borided layer. As in previous parameters, Figure 5b shows the enlarged area from Figure 5a, where the areas from the subsurface (Figure 5c), middle (Figure 6a) and sub-substrate (Figure 6c) of the laser tracks were analyzed. In each of the analyzed areas, a dendritic microstructure can be seen, characterized by very small gaps between the dendrites. This was most likely due to a lower proportion of elements from the substrate. A shorter time of beam interaction on the material contributed to limited remelting and a shallow depth of laser tracks. The areas shown in Figure 5c and Figure 6a,c represent dendrites. They are visible in the transverse and longitudinal sections, depending on the direction of solidification at a given moment in the liquid metal pool. The enlargement of these areas is shown in Figure 5d and Figure 6b,d.

A double increase in scanning speed in relation to the previous sample (v = 50 m/min) resulted in obtaining even smaller remelting. However, no directly proportional reduction in laser track depth was observed. It can be assumed that the scanning speeds applied (25 m/min and 50 m/min) has a limited impact on the diffusion borided layer of high thickness. Such a layer could only melt, but not remelt, with the substrate. A low scanning speed (5 m/min) favors a temperature increase of Monel^®^ Alloy 400 substrate, which, due to good heat capacity of substrate components, results in greater heat accumulation. The use of higher scanning speeds does not contribute to significant heating of the substrate. Figure 7 shows the microstructure of the surface layer following laser processing at a scanning speed of 50 m/min. The microstructure obtained at this speed has a structure similar to that of the aforementioned layer obtained at a scanning speed of 25 m/min. Solidified dendrites related to solidification rate are shown in Figure 7c,d and Figure 8a–d, respectively. The depths of the resulting laser tracks are summarized in Table 2.

Table 3 shows the results of chemical composition analysis using the EDS method for the borided layer and for the laser processed layers. Measurements were made for the areas marked in Figure 2, Figure 3b, Figure 5b and Figure 7b. In the diffusion borided layer, an increased boron content was found in the sub-surface zone. Boron content decreases with increasing distance from the surface, and the values correspond to phases of nickel borides. As a result of laser processing of diffusion boron layer, a remelted zone with a lower boron content was obtained, the amount of which decreased by weight with increasing distance from the surface and with a decrease in laser beam scanning speed. In Table 3, relevant elements in the alloy were taken into account. Table 4 analyses the areas marked in the photos taken at high magnifications (Figure 4b, Figure 6b and Figure 8b). Varying boron content was found in dendritic and inter-dendritic regions, for example, for a layer made at the lowest scanning speed (5 m/min), a high boron content was found in the dendritic areas. With a higher scanning speed, the boron content was comparable. The reason for this was that it was more packed structure. However, it was found that for a scanning speed of 50 m/min a higher weight content of this element was obtained. It was related to the smallest remelting of the diffusion borided layer.

One parameter was selected for further studies of laser processing of the diffusion borided layer. Multiple tracks were made with it. Based on the studies carried out for single tracks (microstructure, microhardness—Section 3.2), it was assumed that the layers produced at a scanning speed of 5 m/min would have the best properties. These parameters resulted in a complete remelting of the diffusion borided layer, and the newly formed surface layer had an even microhardness on the cross-section of laser tracks from the surface into the substrate made of Monel^®^ Alloy 400. Higher scanning speeds (25 m/min and 50 m/min) used in preliminary tests did not ensure complete remelting of the previously obtained diffusion borided layer. As a result, they did not ensure the appropriate thickness of the final coating.

**Figure 9:**
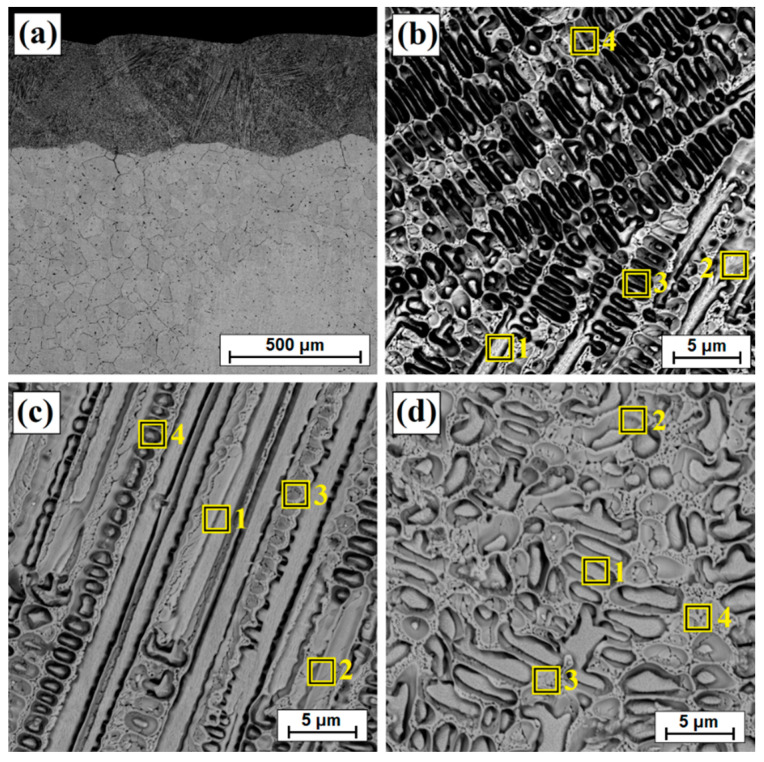
Microstructure of boronized layers after laser processing (v = 5 m/min)—multiple tracks: (**a**) morphology of laser track, (**b**) subsurface zone of laser track, (**c**) middle zone of laser track, (**d**) sub-substrate zone of laser track.

Figure 9 shows the microstructure of the diffusion borided layer after laser processing with a laser beam power of 1400 W, a scanning speed of 5 m/min and a 50% track overlap. These parameters ensured complete filling of the machined surface. It can be seen that the depth of the obtained laser tracks is very similar in the axis and on its boundaries (Figure 9a). A magnified remelted zone from the subsurface, central and near-substrate areas is shown in Figure 9b–d, respectively. In these areas, the sites of chemical composition analysis using the EDS method were marked. As for single tracks, increased boron content was found in dendritic areas. Detailed results of chemical composition measurements are presented in Table 5.

**Figure 10:**
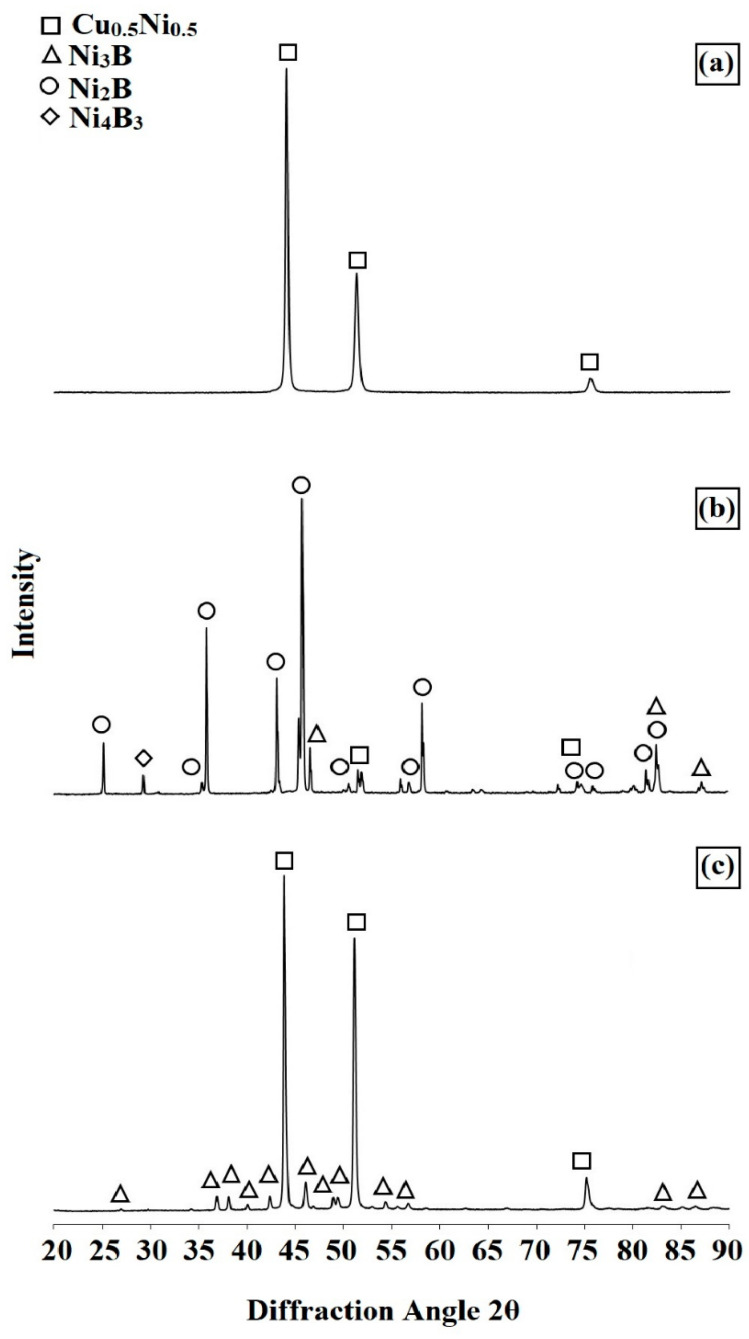
Results of XRD study: (**a**) Monel^®^ Alloy 400 surface, (**b**) diffusion boronized layer, (**c**) diffusion boronized layer after laser processing.

Figure 10 shows the results of phase composition analysis for Monel^®^ Alloy 400 (Figure 10a), and the produced surface layers—diffusion borided (Figure 10b), and laser processed (Figure 10c). Obviously, in a clean substrate a Cu_0.5_Ni_0.5_ solution was detected. In the diffusion borided layer, both the substrate phases (Cu_0.5_Ni_0.5_) and the nickel boride phases, consistent with the Ni-B equilibrium diagram, were identified in X-ray diffraction spectra.

They were Ni_2_B, Ni_3_B and Ni_4_B_3_ borides; an increased intensity of the peaks for the Ni_2_B phase can be noticed. Additional laser processing contributed to a significant domination of the Ni_3_B phase peaks, which confirms boron dilution in the newly formed surface layer.

### 3.2. Microhardness

**Figure 11:**
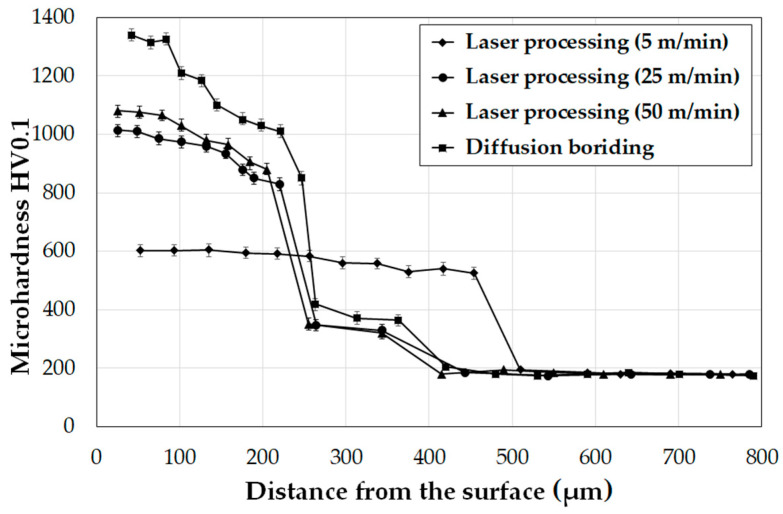
Microhardness of the diffusion boronized layer and single tracks produced by laser processing of diffusion boronized layer formed on Monel^®^ Alloy 400 using a different scanning speed of laser beam.

Figure 11 shows the microhardness profiles of surface layers produced on Monel^®^ Alloy 400. The microhardness of the diffusion and borided layers subjected to laser processing at different scanning speeds was compared. Figure 11 includes only samples on which single tracks were created. The highest microhardness was obtained for the diffusion borided layer. In the area of the needle-like microstructure of boron, it had a value from 1400 HV0.05 to 1100 HV0.05, while in the area under the needles, which was also enriched with boron, a hardness of approximately 400 HV0.05 was obtained. The hardness profile decreases to the hardness corresponding to that of the substrate, i.e., to approximately 200 HV0.05. To compare, in [22], the authors showed that boron layer hardness is related to process parameters and can reach the value of 1050 HV0.05. Mu et al. [19] as a result of pure nickel boriding obtained a hardness of 832–984 HV0.05. On the other hand, Özbek et al. [16] produced nickel borides with a hardness of 747 HV0.05 to 805 HV0.05 in their studies.

**Figure 12:**
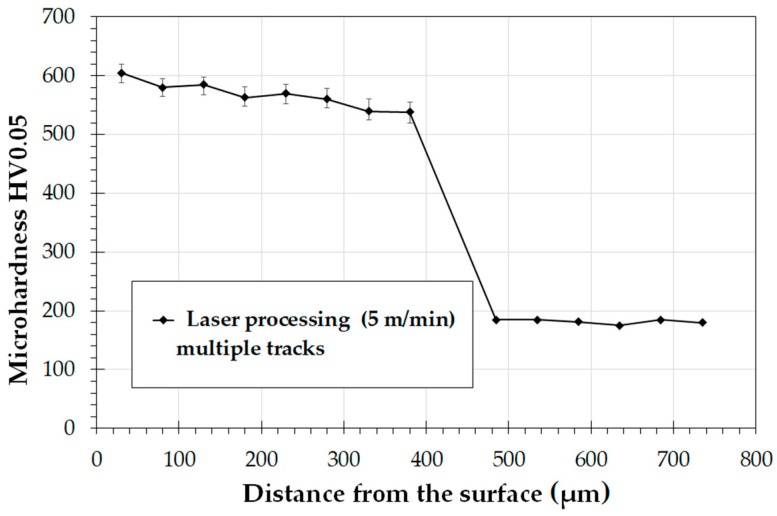
Microhardness of the multiple tracks surface layer produced by laser processing of diffusion boronized layer formed on Monel^®^ Alloy 400.

Laser processing of the boron layer influenced the obtained microhardness, which, depending on the parameters, ranged from 1040 HV0.05 to 600 HV0.05. A low scanning speed v = 5 m/min contributed to obtaining laser tracks twice as thick as in diffusion-borided layer, but at the same time with a hardness twice as low as in this layer. However, the surface layer obtained was characterized by a uniform microhardness over the entire cross-section of the laser track. At the same time, the hardness of the layer was three times higher than that of the substrate of Monel^®^ Alloy 400. The use of higher scanning speeds with the laser beam of 25 m/min or 50 m/ min contributed to an increase in microhardness by 5–6 times compared to the substrate material. However, as a result of laser beam modification, the entire diffusion-borided layer did not remelt. The use of high scanning speeds did not extend the area of boron appearance but only remelted the material in the needle-like boride zone. The decrease in hardness in the remelted zone as compared to the boride layer was related only to an increased substrate share. However, the aim of the study was to create a layer that will have a hardness greater than the substrate and will be much thicker than the boride layer. Hence the choice of the scanning speed v = 5 m/min. Figure 12 shows the microhardness profile of the laser diffusion modified boride layer at a scanning speed of 5 m/min. The results are averaged values of microhardness measurements in the axes of 10 tracks. It can be seen that microhardness of the newly formed layer in all created laser tracks was comparable and was in the range of approximately 600 HV0.05. Laser beam scanning speed used as well as laser track overlap of 50% (f = 0.5 mm) ensured a layer with a uniform thickness along the axis and on laser track boundaries.

### 3.3. Wear Resistance

**Figure 13:**
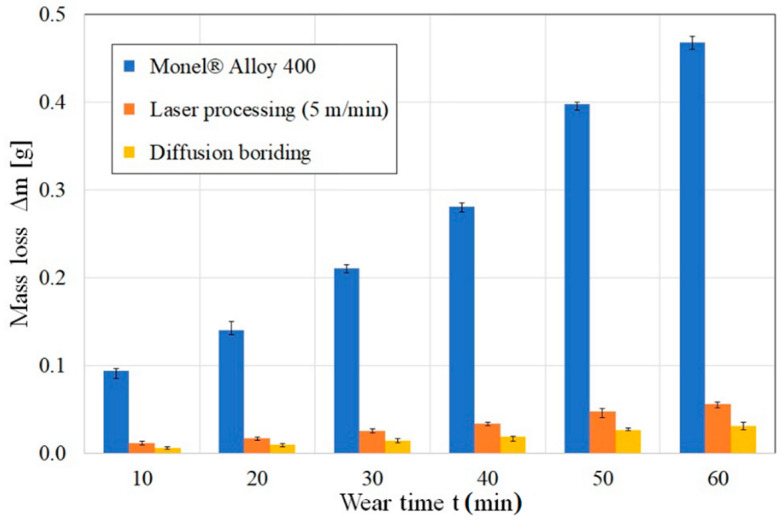
Values of mass loss obtained during wear resistance tests of the Monel^®^ Alloy 400 before and after various type modifications.

Figure 13 shows the results obtained after wear resistance tests. Both untreated substrate material and the produced surface layers were tested. The graph shows weight loss expressed in grams. On the basis of the conducted analysis it was found that the production of surface layers significantly increases friction wear resistance. The surface layer produced by diffusion boriding showed better wear resistance. However, this layer is thin. Laser processing allowed for a significant increase in wear resistance compared to pure material Monel^®^ Alloy 400. According to the authors, the surface layer obtained by laser processing of a diffusion borided layer is a compromise between a very hard diffusion layer of small thickness and the complete absence of a layer. Increasing the thickness of the surface layer will significantly extend the life of the product on which it will be produced. The only condition is a regime of maintaining the dimensional stability of a given product.

**Figure 14:**
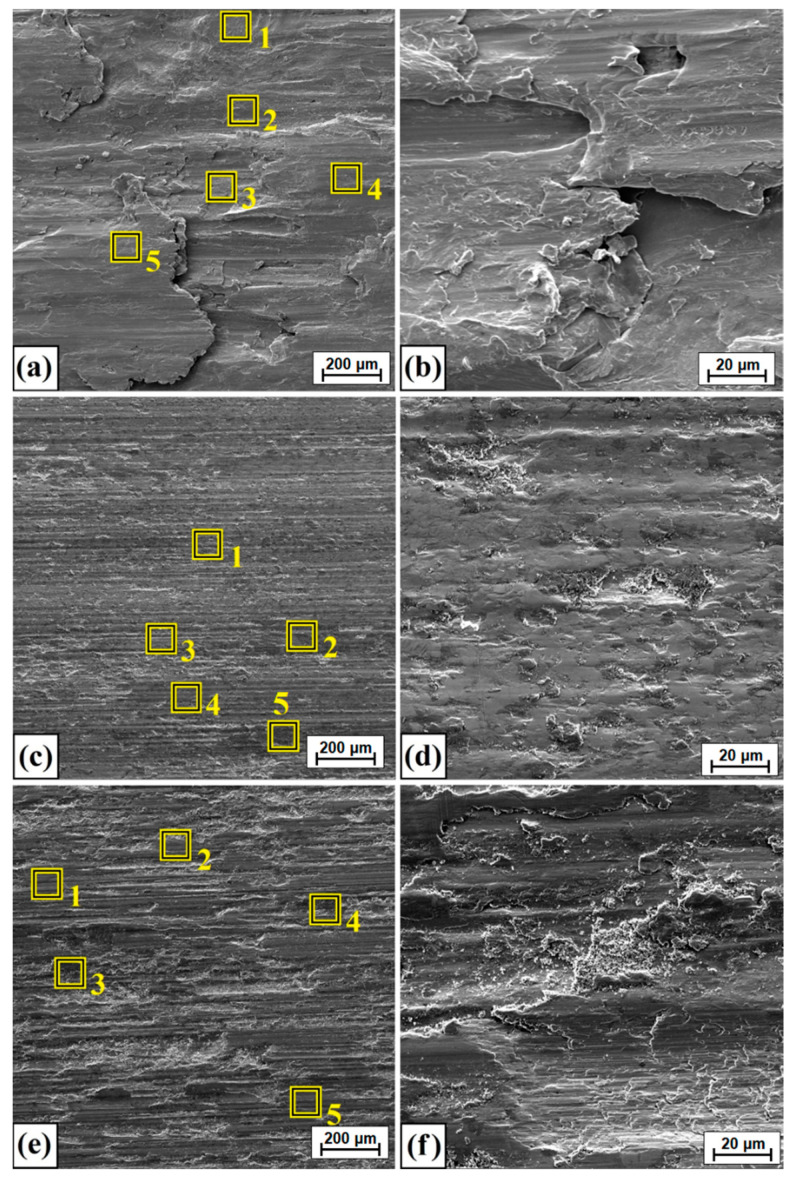
Surface condition after wear resistance tests: (**a**) Monel^®^ Alloy 400 surface, (**b**) magnification of Monel^®^ Alloy 400 surface, (**c**) diffusion boronized layer, (**d**) magnification of diffusion boronized layer, (**e**) diffusion boronized layer after laser processing, (**f**) magnification of diffusion boronized layer after laser processing.

Figure 14 shows the surface following friction wear tests. Figure 14a shows the worn surface of Monel^®^ Alloy 400. A characteristic type of adhesive wear can be seen. To confirm this type of wear, a magnification of the area shown in Figure 14a is presented in Figure 14b. Additionally, to analyze changes in chemical composition, selected areas subject to analysis are marked in Figure 14a, and the results are summarized in Table 6. In diffusion borided and laser processed samples, a dominant mechanism is abrasive–adhesive wear. Here, too, the magnified areas of Figure 14c,e are shown in Figure 14d,f for diffusion borided Monel, and Monel borided following laser processing, respectively. The results of chemical composition tests using the EDS method are presented in Table 6.

**Figure 15:**
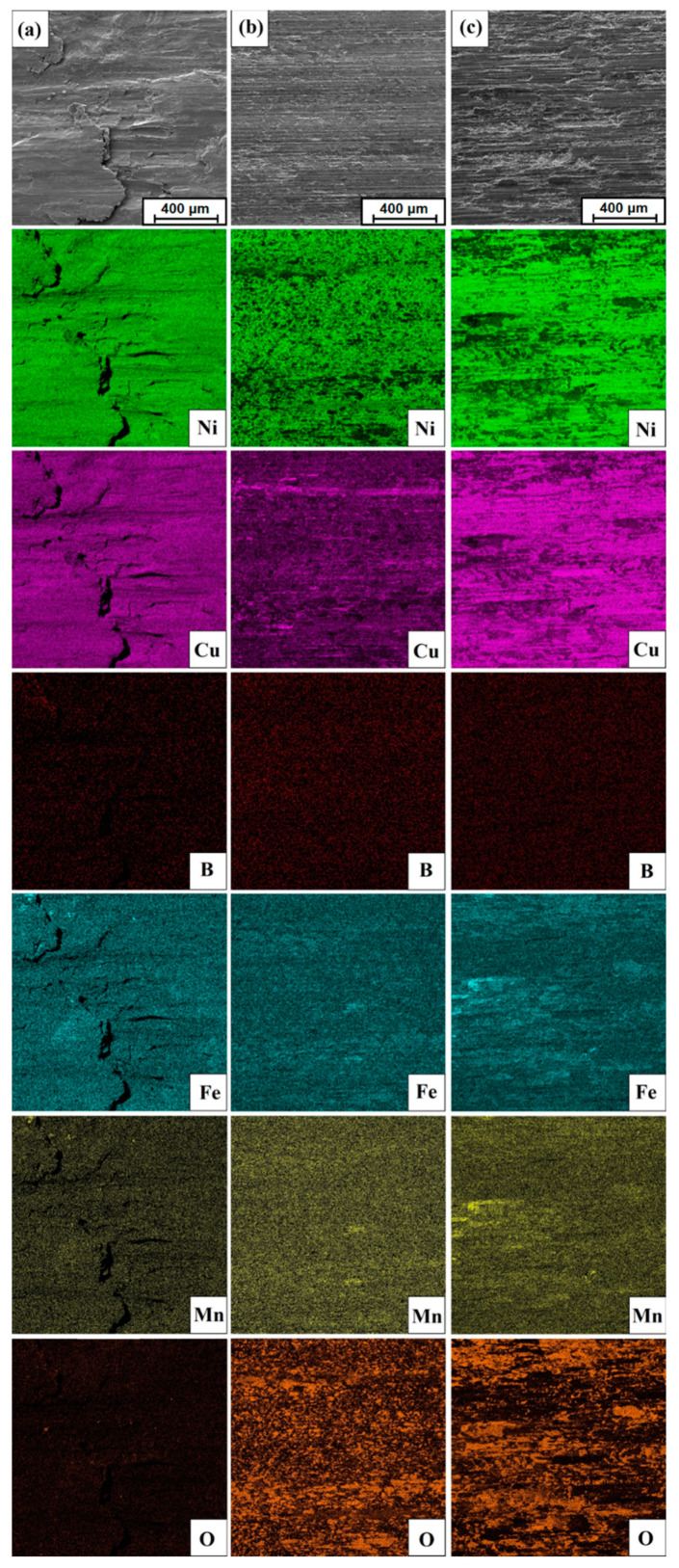
EDS mapping of surface condition after wear resistance tests: (**a**) Monel^®^ Alloy 400 surface, (**b**) diffusion boronized layer, (**c**) diffusion boronized layer after laser processing.

It can be seen that the surfaces of samples with defined surface layers oxidized the most. Iron from a steel counter-sample was also observed on their surface. This is due to the greater hardness of surface layers than that of the pure substrate. In addition, for the analyzed surfaces of the post-wear samples, chemical composition maps were made using the EDS method, where it is possible to observe the sites of oxygen accumulation caused by oxidation and iron from the counter-sample (Figure 15).

**Figure 16:**
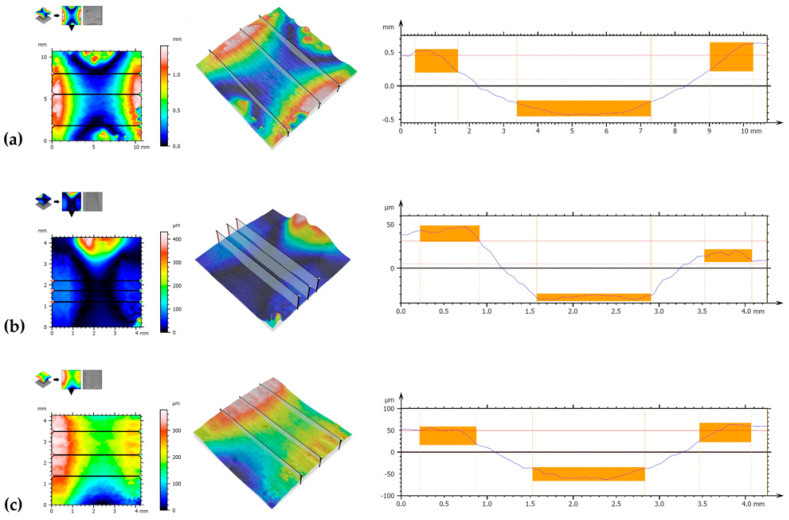
3D images surface condition for: (**a**) Monel^®^ Alloy 400 surface, (**b**) diffusion boronized layer, (**c**) diffusion boronized layer after laser processing.

Figure 16 shows the surface topography of samples following wear resistance tests. The first image shows SEM image necessary for a 3D analysis of the image surface. The photos were taken at an angle of +4° and −4°. Depending on their topographic contrast, a stereoscopic reconstruction of the created layer was performed. Figure 16 also shows the places where surface wear profiles were made. Detailed measurement results obtained for samples used are summarized in Table 7. Selected parameters of 2D and 3D surface roughness were analyzed. From the group of 2D parameters, parameters *Ra* (deviation of arithmetic mean of the profile) and *Rz* (maximum height of the roughness profile) were analyzed. However, from the group of 3D parameters, *Sa* (arithmetic mean deviation of the surface unevenness height from the reference plane), *Sq* (mean square deviation of surface unevenness height from reference planes) and *Sz* (maximum height of 3D profile) were analyzed. Besides 3D photos, Figure 16 shows one of the profiles manifesting traces of wear following tribological tests. A diffusion-borided layer and a laser processed layer are characterized by a comparable width of the wear mark, but the diffusion-borided layer has the smallest depth of this mark. The highest wear trace was observed for a pure substrate without a surface layer formed. Surface roughness in 2D system for diffusion borided layer as well as for the laser borided layer is similar and is 1.697 µm and 2.011 µm for the *Ra* parameter, respectively.

### 3.4. Corrosion Resistance

**Figure 17:**
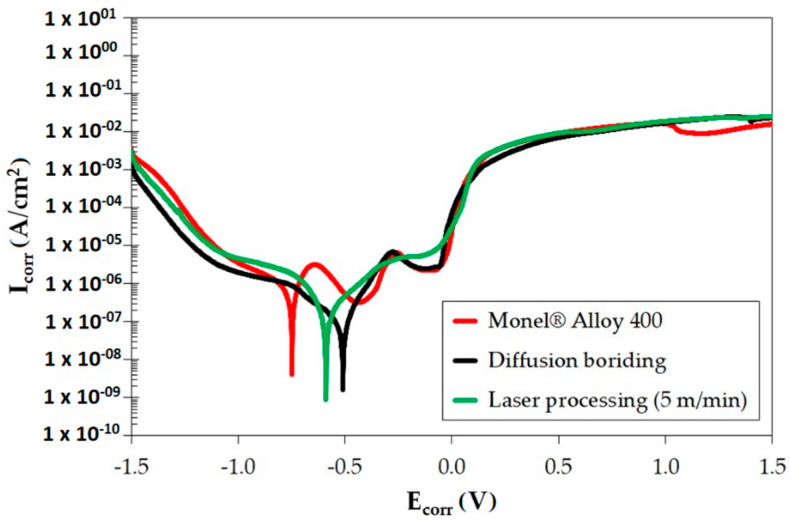
Corrosion resistance tests results for substrate material (Monel^®^ Alloy 400 surface), diffusion boronized layer and diffusion boronized layer after laser processing.

Results of the corrosion resistance test are shown in Figure 17. It can be seen from the presented graph that diffusion borided layer is characterized by the best corrosion resistance. Laser treatment slightly worsens corrosion resistance. However, modifying the Monel surface with diffusion and laser diffusion methods improves the resistance of this type of alloy. Detailed results for corrosion current and corrosion potential are summarized in Table 8.

**Figure 18:**
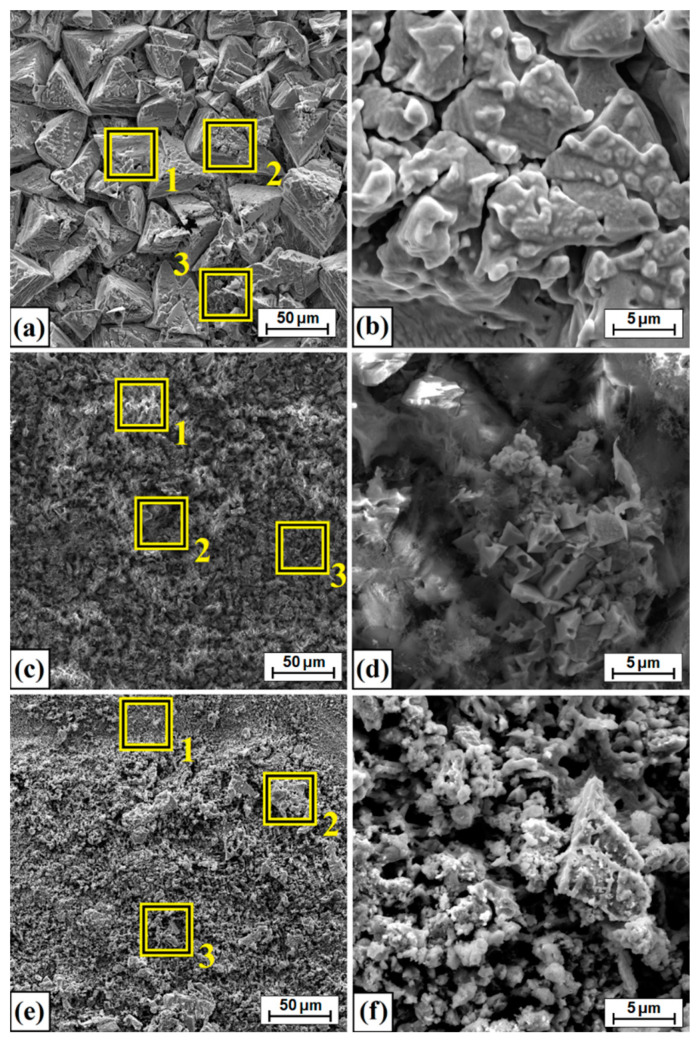
Surface condition after corrosion resistance tests: (**a**) Monel^®^ Alloy 400 surface, (**b**) magnification of Monel^®^ Alloy 400 surface, (**c**) diffusion boronized layer, (**d**) magnification of diffusion boronized layer, (**e**) diffusion boronized layer after laser processing, (**f**) magnification of diffusion boronized layer after laser processing.

**Figure 19:**
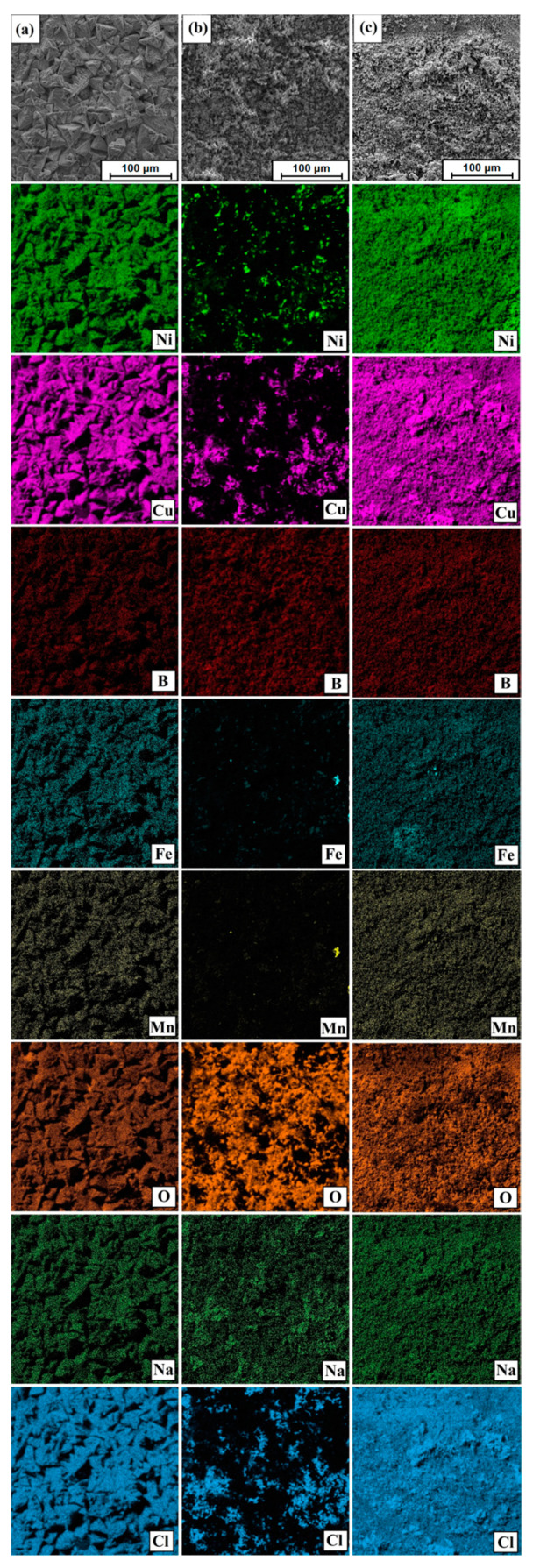
EDS mapping of surface condition after corrosion resistance tests: (**a**) Monel^®^ Alloy 400 surface, (**b**) diffusion boronized layer, (**c**) diffusion boronized layer after laser processing.

Figure 18 shows the surface condition after testing corrosion resistance of Monel^®^ Alloy 400 before and after the surface layer fabrication process. As in the case of wear resistance tests, comprehensive chemical composition tests were carried out using the EDS method. Figure 18a,c,e show the sites where the areas were analyzed by the EDS method. Magnified areas of corrosive medium impact are shown in Figure 18b,d,f for the pure substrate, not subjected to any modification, the diffusion borided layer and the layer produced as a result of laser processing of the boron layer, respectively. The EDS tests results for the areas marked in Figure 18 are shown in Table 9, while the EDS mapping of the surface after the corrosion resistance tests is shown in Figure 19. It can be seen that oxygen and chlorine are unevenly distributed on the borided sample. In sites where chlorine accumulates, there is no oxidized surface, and vice versa. However, in a layer borided after laser processing, the difference in the intensity of the elements that play a significant role in the corrosion process is very similar, which proves their uniform distribution in the analyzed site.

## 4. Conclusions

The study analyzed the influence of surface modification of Monel^®^ Alloy 400 material on the properties obtained. A diffusion borided layer was produced and then processed with a diode laser. Following conclusions were drawn from the studies:

1. A diffusion borided layer produced on Monel^®^ Alloy 400 was characterized by fine needle-like microstructure of nickel borides at the surface, under which there was a solution zone enriched with boron.
2. Microhardness of the diffusion boron layer ranged from 1400 HV0.05 in the sub-surface zone to 1100 HV0.05 in the zone near the substrate. Laser processing contributed to microhardness reduction. It was found that microhardness decreased with a decrease in scanning speed. Using the highest scanning speed, a microhardness of around 1050 HV0.05 was achieved, while at the lowest scanning speed of 600 it was HV0.05.
3. As a result of remelting the borided layer with a laser beam, the microstructure of the remelted zone was composed of boron-nickel eutectic with nickel boride phases Ni_3_B, Ni_2_B and Ni_4_B_3_.
4. Both diffusion boriding and laser modification contributed to increased corrosion resistance and friction wear resistance compared to the properties of untreated Monel^®^ Alloy 400 material.
5. Laser processing of the diffusion borided layer on Monel^®^ Alloy 400 leads to a reduction in mechanical and physicochemical properties (corrosion resistance, wear resistance) compared to the diffusion borided layer. It should be emphasized, however, that the boron layer after laser processing was much thicker than the diffusion layer. At the same time, such a layer is less prone to cracking due to lower brittleness, which means that it can be used in selected applications.

## Figures and Tables

**Table 1:** Chemical composition of Monel^®^ Alloy 400 material used (wt.%).

Cu	Si	Fe	Mn	C	S	Ni
31	0.5	2.5	2.0	0.3	0.024	bal.

**Table 2:** Thicknesses of surface layers produced by laser processing of diffusion boronized layers [µm].

Scanning Speed of Laser Beam	Single Track	Multiple Tracks	
		In the Axes of the Laser Tracks	On Border of the Laser Tracks
v = 5 m/min	600	459	370
v = 25 m/min	212	no tested	
v = 50 m/min	150	no tested	

**Table 3:** Chemical composition study results (EDS) of surface layers produced by diffusion boronizing and using laser processing of diffusion boronized layers (wt.%).

Type of Layer	No	Ni	Cu	B	Fe	Mn
Diffusion boronized	1	86.8	2.1	7.7	2.7	0.6
	2	86.0	2.9	7.1	3.6	0.4
	3	86.1	4.5	6.2	2.7	0.5
	4	86.4	5.0	5.3	3.0	0.3
Laser processing of diffusion boronizing layer v = 5 m/min	1	82.0	9.8	5.2	2.6	0.4
	2	81.5	10.8	4.7	2.4	0.6
	3	85.8	6.7	5.2	2.1	0.2
	4	85.6	6.3	4.7	3.0	0.4
	5	67.3	28.2	1.2	2.5	0.8
	6	65.4	30.8	0.5	2.2	1.1
Laser processing of diffusion boronizing layer v = 25 m/min	1	80.2	10.0	6.6	2.7	0.6
	2	81.2	9.6	6.4	2.4	0.3
	3	80.1	11.7	5.5	2.4	0.3
	4	87.8	5.4	3.8	2.5	0.5
	5	89.3	4.4	3.7	2.4	0.3
	6	64.8	33.1	0.0	1.4	0.7
Laser processing of diffusion boronizing layer v = 50 m/min	1	81.4	9.7	6.8	2.1	0.1
	2	82.3	8.7	6.5	2.3	0.2
	3	87.3	2.9	7.3	2.4	0.2
	4	82.7	8.8	5.9	2.4	0.2
	5	87.5	4.8	4.9	2.4	0.4
	6	66.7	30.1	0.0	2.0	1.2

**Table 4:** Chemical composition study results of surface layers produced by laser processing of diffusion boronized layers obtained using EDS method (middle zone of laser track) (wt.%).

Scanning Speed of Laser Beam	No	Ni	Cu	B	Fe	Mn
v = 5 m/min	1	84.6	7.8	4.1	2.8	0.7
	2	85.5	7.6	3.8	2.4	0.8
	3	87.9	5.4	3.9	2.2	0.5
	4	85.5	7.5	4.4	2.1	0.6
v = 25 m/min	1	77.6	13.8	6.1	2.1	0.4
	2	80.9	10.8	6.1	2.1	0.2
	3	78.0	13.3	5.7	2.4	0.6
	4	81.2	10.7	5.6	2.4	0.1
v = 50 m/min	1	80.5	9.0	7.5	2.7	0.2
	2	79.4	11.2	6.9	2.3	0.2
	3	85.2	5.4	7.2	2.2	0.2
	4	81.7	9.1	6.8	2.0	0.3

**Table 5:** Chemical composition study results of surface layer produced by laser processing of diffusion boronized layer (v = 5 m/min) obtained using EDS method (wt.%).

Zone of Layer	No	Ni	Cu	B	Fe	Mn
Subsurface zone of laser track	1	81.8	10.7	5.3	1.5	0.6
	2	86.6	7.0	4.2	1.7	0.5
	3	88.0	6.8	3.5	1.1	0.5
	4	87.1	7.3	3.5	1.5	0.6
Middle zone of laser track	1	84.2	8.2	5.9	1.3	0.3
	2	79.0	13.1	5.5	1.7	0.7
	3	84.3	8.1	4.7	2.3	0.7
	4	88.2	5.7	4.2	1.5	0.5
Sub-substrate zone of laser track	1	89.3	6.1	2.1	1.8	0.8
	2	87.0	7.6	3.0	2.0	0.4
	3	89.6	4.9	3.8	1.2	0.5
	4	89.4	4.9	3.9	1.4	0.3

**Table 6:** Chemical composition study results after wear resistance tests obtained using the EDS method (wt.%).

Type of Layer	No	Ni	Cu	B	Fe	Mn	O
Monel^®^ Alloy 400 (substrate material)	1	61.6	32.4	-	4.2	1.2	0.6
	2	58.7	32.6	-	7.3	0.7	0.7
	3	62.0	34.0	-	2.5	1.0	0.4
	4	66.4	29.4	-	2.9	0.9	0.5
	5	60.8	32.1	-	5.9	0.7	0.6
Diffusion boronzing	1	71.8	20.1	1.4	4.1	1.0	1.6
	2	55.1	25.5	0.1	13.5	0.6	5.3
	3	21.2	12.8	0.0	44.4	0.6	20.9
	4	25.1	15.5	0.0	39.8	0.9	18.7
	5	22.4	13.7	0.0	44.1	0.6	19.1
Laser processing of diffusion boronizing layer	1	32.0	16.4	0.0	38.5	0.7	12.5
	2	64.5	29.7	0.0	3.6	0.9	1.4
	3	35.1	17.9	0.0	30.4	0.6	15.9
	4	63.9	30.0	0.0	3.7	1.1	1.3
	5	65.4	28.7	0.4	4.0	0.5	1.0

**Table 7:** Surface roughness parameters for selected profile obtained during tests (µm).

Parameters	Monel^®^ Alloy 400 (Substrate Material)	Diffusion Boronizing	Laser Processing of Diffusion Boronizing Layer
*Ra*	4.746	1.697	2.011
*Rz*	18.23	7.713	8.015
*Sa*	309.9	45.69	45.77
*Sq*	363.9	66.11	53.82
*Sz*	1422	444.5	234.9
wear track width	3908.0	1327	1306
max. wear track depth	889.0	67.48	112.3
average wear track depth	828.3	64.46	102.8

**Table 8:** Corrosion current and corrosion potential for substrate material (Monel^®^ Alloy 400 surface), diffusion boronized layer and diffusion boronized layer after laser processing.

Specimen	Current *I*_corr_ [A·cm^2^]	Potential *E*_corr_ [V]
Monel^®^ Alloy 400	3.54 × 10^−7^	−7.48 × 10^−1^
Diffusion boronizing	4.07 × 10^−8^	−5.09 × 10^−1^
Laser processing of diffusion boronizing layer	5.54 × 10^−6^	−5.97 × 10^−1^

**Table 9:** Chemical composition study results after corrosion resistance tests obtained using EDS method (wt.%).

Process	No	Ni	Cu	O	Cl
Monel^®^ Alloy 400 (substrate material without modification process)	1	14.7	45.9	17.6	21.8
	2	25.1	39.2	13.4	22.3
	3	11.1	39.6	7.7	41.7
Diffusion boronzing	1	87.4	9.8	2.4	0.5
	2	9.8	20.6	62.3	7.2
	3	38.9	8.1	51.8	1.2
Laser processing of diffusion boronizing layer	1	2.2	51.2	15.9	30.9
	2	2.7	52.6	13.7	31.1
	3	1.4	57.9	5.9	34.8

## Data Availability

Data available on request.
